# Prescriptive Appropriateness and Efficacy of Cholesterol-Lowering Drugs in a Secondary Prevention Setting—A Retrospective Analysis from Two Italian Cardiac Rehabilitation Centers

**DOI:** 10.3390/jcm13247505

**Published:** 2024-12-10

**Authors:** Francesca Saladini, Stefania Baggio, Federica Marcato, Francesco Campisi, Roberto Verlato, Giampaolo Pasquetto, Emanuele Bertaglia, Gaetano Povolo, Paolo Buja, Nicola Ferri

**Affiliations:** 1 Cardiology Unit, Cittadella Town Hospital, 35013 Cittadella, Italy; roberto.verlato@gmail.com (R.V.); giampaolo.pasquetto@aulss6.veneto.it (G.P.); gaetano.povolo@aulss6.veneto.it (G.P.); paolo.buja@aulss6.veneto.it (P.B.); 2 Pharmacology Unit, Camposampiero Town Hospital, 35012 Camposampiero, Italy; stefania.baggio@studenti.unipd.it (S.B.); federica.marcato@aulss6.veneto.it (F.M.); 3 Cardiology Unit, Camposampiero Town Hospital, 35012 Camposampiero, Italy; francesco.campisi@aulss6.veneto.it (F.C.); emanuele.bertaglia@aulss6.veneto.it (E.B.); 4 Pharmacology, University of Padova, 35131 Padova, Italy; nicola.ferri@unipd.it

**Keywords:** statin, cardiovascular disease, dyslipidemias, adherence to treatment, cardiac rehabilitation

## Abstract

**Background:** Treatment of CV risk factors, such as cholesterol level, represents one of the main goals to reduce atherosclerotic burden. The aim of this study was to investigate the prescriptive appropriateness of cholesterol-lowering drugs among patients who experienced an atherosclerotic CV disease (ASCVD). **Methods**: We investigated 155 patients who underwent cardiac rehabilitation in 2020. The European Society of Cardiology (ESC) 2021 guidelines on CV disease prevention and 2019 ESC Guidelines on dyslipidemias were followed to detect the appropriateness of prescription. SCORE2 and SCORE2-OP risk estimations were used to detect patients’ CV risk profiles. Patients were divided into three groups: 1 (n = 118) patients admitted for their first CV event, 2A (n = 18) patients who experienced a previous CV event years before, and 2B (n = 19) patients admitted for a new event with a previous CV event 2 years before. Low-density lipoprotein (LDL) cholesterol level was detected at the time of admission to the hospital, during cardiac rehabilitation, and at the first visit after rehabilitation. **Results:** The statistics for our study participants, with a mean age of 66.1 years, were: 72.4% overweight/obese, 63.9% diabetic, 72.5% smokers, 93.0% hypertensives, and 91.7% had dyslipidemias. In group 1, only 5.1% had a low/moderate risk, 44.1% presented a high risk, and 50.8% a very high risk according to calculators. The average LDL levels were 115.8 mg/dL (2.99 mol/L) upon admission to the hospital, 66.4 mg/dL (1.72 mmol/L) at the time of cardiac rehabilitation, and 64.8 mg/dL (1.67 mmol/L) at the subsequent medical visit. In the overall group, only 36.0% had LDL < 55 mg/dL (1.42 mmol/L). In group 1, 79.4% were treated with high-intensity statin alone or plus ezetimibe; in group 2A, the percentage increased up to 87.5%, while group 2B 33.4% was treated with high-intensity statin plus ezetimibe and 33.3% were treated with PCSK9 inhibitors. **Conclusions**: This retrospective study confirms the importance of properly calculating CV risk profiles. The main limitations for the efficacy of lipid-lowering drugs were: patient’s compliance, drugs side effects, lifestyle habits, and collaboration with a general practitioner.

## 1. Introduction

According to the World Health Organization, cardiovascular (CV) diseases still represent the leading cause of death among the non-communicable diseases all around the world [1]. It was observed that, in 2017, CV diseases were responsible for 17.8 million deaths, and the majority occurred in low-income and middle-income countries [2]. Also, in Europe, CV diseases represent one of the main causes of death, being responsible for more than 4 million deaths (45% of all causes of death) [3], as well as in Italy, where, according to the Istituto Superiore di Sanità data, CV diseases are the more prevalent causes of death, being responsible for 44% deaths. In particular, 28% is due to myocardial infarction and 13% to cerebrovascular events (the third cause of death after malignancy).

Several risk factors contribute to the development of atherosclerotic CV diseases (ASCVD), like smoking habits, overweight and obesity status, sedentary lifestyle, high blood pressure (BP) values, and high levels of serum glucose and cholesterol, and the main goals for the prevention of CV events are the promotion of a healthy lifestyle and the correction of risk factors [4]. Among these, one of the most challenging tasks is the reduction in cholesterol level and, in particular, low-density lipoprotein (LDL) reduction, as these are the underlying causes of the atherosclerosis process, and its reduction is strictly correlated to the reduction in CV diseases [5,6]. According to this evidence, several recent guidelines proposed more severe goals for targeting LDL [4,5]. The European Society of Cardiology (ESC) guidelines for the prevention for CV disease [4] proposed a threshold level < 55 mg/dL (<1.4 mmol/L) plus a reduction of 50% of the initial level for patients with a very high CV risk, and a more ambitious target of <40 mg/dL (<1.0 mmol/L) for patients with ASCVDs who experienced a second CV event within 2 years [5] of the previous one. These lower levels were suggested according to the evidence from a recent metanalysis that showed that the risk reduction in ASCVD is directly correlated with the absolute reduction in LDL levels [5]. In particular, it was observed that every 1 mmol/L of absolute decrease in LDL by statins led to a reduction in the all-cause mortality of 10% and of 22% for major adverse CV events [7]. To reach these ambitious targets, both guidelines [4,5] proposed a detailed step-by-step approach that included improved lifestyle habits and lipid-lowering drugs as monotherapy or as combination therapy with ezetimibe and/or protein convertase subtilisin/kexin type 9 (PCSK9) inhibitors. Unfortunately, as documented by Da Vinci’s [8] and Santorini’s [9] studies, clinical practice for the management of lipid-lowering therapy both in high- and very high-risk patients, either for primary or secondary prevention, was far from reaching the threshold levels suggested in the latest guidelines [4,5]. The failure to achieve the suggested LDL goals was explained by the gap in the knowledge of physicians regarding the new guidelines; the underestimation of the true risk profile of the patients; the underprescription of combination therapy; the high costs of new therapies, such as PCSK9 inhibitors; and the reluctance of patients to accept lipid-lowering drugs, due to the possible adverse effects, especially of statins.

The aim of the present study was to retrospectively investigate the clinical practice for evaluations of the cardiovascular risk profile, appropriate prescription of lipid-lowering drugs, and LDL goals reached in a secondary prevention setting among patients hospitalized for an ASCVD and subsequently followed during cardiac rehabilitation at the Cittadella and Camposampiero hospitals in Azienda ULSS 6 Euganea, in Padova, in 2020. The prescriptive appropriateness was investigated by clinical audit.

## 2. Materials and Methods

This retrospective study examined all medical records available for patients who underwent cardiac rehabilitation in the Cittadella and Camposampiero town hospitals in 2020 after an ASCVD. From the initial number of 175 patients that attended the two cardiac rehabilitations, during the period considered, 20 patients were excluded because they attended rehabilitation after an acute event different from an ASCVD (for example, surgery for a valve replacement/reparation or advanced heart failure without the association coronary artery disease). Finally, we included data from 155 patients for whom electronic medical records were available, at the time of admission to hospital, during cardiac rehabilitation, and at the first medical visit available after cardiac rehabilitation, within 1 year after discharge from cardiac rehabilitation. The data gathered included anthropometric parameters, medical history, BP levels, lipid profile, and drugs prescribed. Patients included were all patients with an ASCVD with a very high CV risk, according to the following guidelines: 2021 ESC Guidelines on CV disease prevention in clinical practice [4] and 2019 ESC/EAS Guidelines for the management of dyslipidemias: lipid modification to reduce cardiovascular risk [5], those who should reach the LDL goal of <55 mg/dL (<1.4 mmol/L) and LDL reduction of 50% from the baseline value or <40 mg/dL (<1.03 mmol/L) in the case of a second CV event within 2 years of the previous one. The presence of hypertension was detected by the assumption of anti-hypertensive drugs or according to office BP values ≥ 140/90 mmHg [10]. The presence of diabetes mellitus was defined according to the presence of hypoglycemic drugs, previous diagnosis of diabetes mellitus, or the presence of elevated plasma glucose levels according to the guidelines [11]. Dyslipidemia was detected by the use of lipid-lowering drugs, medical history of dyslipidemia, or LDL level above the threshold level suggested, according to the individual CV risk profile [5]. Overweight status was defined as a body mass index (BMI) ranging from 25 to 29.9 kg/m^2^ and obesity as a BMI ≥ 30 kg/m^2^ or higher [12]. The classification as high-intensity or moderate–low-intensity statins is reported in Table 1.

### 2.1. Cardiovascular Risk Estimation

According to the medical records obtained from the electronic data, the CV risk profile of the patient was obtained by Systematic Coronary Risk Estimation 2 (SCORE2) and Systematic Coronary Risk Estimation 2—Older Persons (SCORE2-OP) risk charts for individuals with a 10-year risk of fatal and non-fatal (myocardial infarction and stroke) CV disease events in a population with a moderate CV disease risk, such as Italy, according to the latest guidelines [4]. These calculators take into account gender, smoking status, age, systolic BP level, and non-HDL cholesterol level [4].

### 2.2. Cardiac Rehabilitation

Cardiac rehabilitation lasted for 8 medical sessions, was performed 3 times per week, and was arranged 4 to 12 weeks after acute CV events had passed, according to the guidelines [13]. Patients were followed by cardiologists and nurses, experts in cardiac rehabilitation, and were exposed to clinical examinations, BP measurements, and biochemistry, including evaluations of their lipid profiles. BP was measured three times, with the patient in a sitting position, by a validated device, according to the guidelines [14], at the start of their cardiac rehabilitation. During each cardiac rehabilitation session, BP was taken at the beginning and at the end of exercise. For each patient, a personalized lifestyle intervention program and exercise program were suggested during the training period, according to the guidelines [13]. Each cardiac rehabilitation session included an educational session with reinforcement for lifestyle improvement, BP measurements, an exercise training session, and a cooldown phase. At the end of cardiac rehabilitation, the cardiologist planned a follow-up visit within 1 year, with an updated lipid profile. The inclusion and exclusion criteria for cardiac rehabilitation are listed in Appendix A.

### 2.3. Clinical Audit

According to the procedure described by Bampton et al. [15], we performed clinical audits in three different sessions, examining the electronic data regarding the time of CV event, data regarding cardiac rehabilitation, and data collected at the time of the first follow up. The discussion was about the following issues: the appropriateness of the prescription at the time of discharge from the hospital and of discharge from cardiac rehabilitation; definition of the standard of care in this particular subset of patients according to the clinical guidelines [4,5]; comparison between standards of care and clinical practice; identification of inappropriateness of care; suggestions of means to improve and facilitate appropriateness of care; and new clinical audits to verify improvements (this last part is still ongoing).

All patients provided written informed consent, and the study followed the ethical standards as presented in the 1964 Declaration of Helsinki and its later amendments.

### 2.4. Statistical Analysis

Quantitative variables were reported as mean ± standard deviation and the differences in the distribution across groups were tested by one-way ANCOVA analysis adjusted for age and sex. The only non-normally distributed variable was triglyceride level, and the *p* value for this parameter was reported for log-transformed data. Categorical variables were reported as percentages and the chi-squared distribution was used to compare the variables. LDL cholesterol was determined from serum analysis, and if it was not available, it was calculated by means of the Friedewald formula [5]. For the analysis, patients were divided into three subgroups: 1 (n = 118)—patients admitted for their first CV event; 2A (n = 18)—patients who experienced a previous CV event and were admitted to hospital following a new event, years after the previous one; and 2B (n = 19)—patients who experienced a new CV event within 2 years of the previous one. A 2-tailed probability value < 0.05 was considered significant. All analyses were performed using Systat version 12 (SPAA Inc., Evanston, IL, USA).

## 3. Results

The study participants included 155 subjects who underwent cardiac rehabilitation at Cittadella or Camposampiero town hospital (Padova) in 2020. The mean age of the study participants was 66.1 ± 9.4 years, and the vast majority were males (88.4%). The prevalence of comorbidities is reported in Table 2. Of note, there was a high prevalence of overweight and obesity status, dyslipidemias, and hypertensives, and more than two thirds of the study participants were smokers. Of note, comorbidities did not differ according to the subgroups.

Metabolic and hemodynamic characteristics at entry are described in Table 3. Serum glucose and BMI did not differ significantly among groups, while total cholesterol and LDL cholesterol were significantly higher among those in group 1 in comparison to those who experienced a previous CV event (Table 3). Systolic BP as well as diastolic BP were higher among those admitted for their first CV event, but the differences did not reach the level of statistical significance (Table 3; in Table A1, the data are presented as means and IQRs).

**Figure 1:**
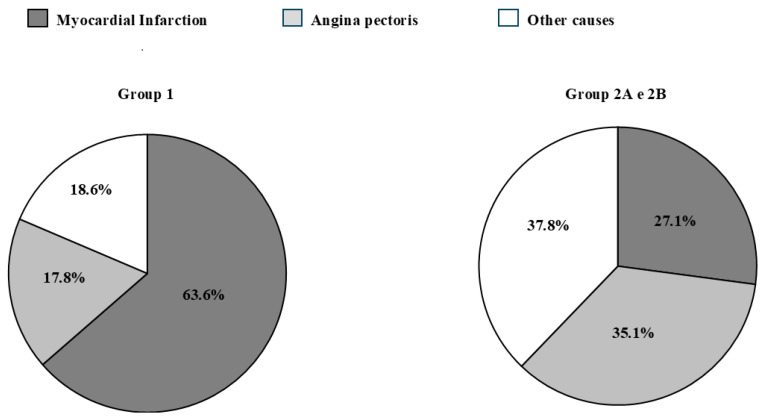
Reasons for admission to hospital among group 1 and group 2A and 2B patients considered as a whole group.

In particular, among those who were admitted for the first time for the occurrence of a CV event, according to the SCORE2 and SCORE2-OP risk charts, only 5.1% had a low/moderate risk, while the vast majority was classified as having a high risk (44.1%) or very high risk (50.8%) of experiencing fatal or non-fatal CV events in the following 10 years. Of note, at entry, among this group, the vast majority (81.3%) was not treated with lipid-lowering drugs, despite the high-risk profile; 11.9% were taking low-intensity statins; 1.7% were taking high-intensity statins; 0.8% were taking a single-pill combination of statin plus ezetimibe; 0.8% were taking ezetimibe alone; and 3.5% were taking red yeast rice or other nutraceutical products, with low efficacy on cholesterol treatment, as LDL cholesterol at baseline was 115.8 ± 39.4 mg/dL (2.99 ± 1.02 mmol/L) in the whole group and 122.8 ± 38.2 mg/dL (3.17 ± 0.99 mmol/L) in group 1 study participants. The reasons for admission to hospital for group 1 and group 2A and 2B participants are reported in Figure 1: the most frequent reason for admission among group 1 patients was myocardial infarction, while among groups 2A and B subjects, myocardial infarction was less frequent and the main reasons were other causes with an associated increase in cardiac serum markers, such as heart failure, atrial fibrillation, or angiographic control of known coronary artery diseases (the complete list of events according to the subgroups is presented in Appendix B).

LDL cholesterol levels at admission to hospital, at the time of cardiac rehabilitation, and at the first control visit within 1 year of follow up after cardiac rehabilitation are reported in Table 4.

**Figure 2:**
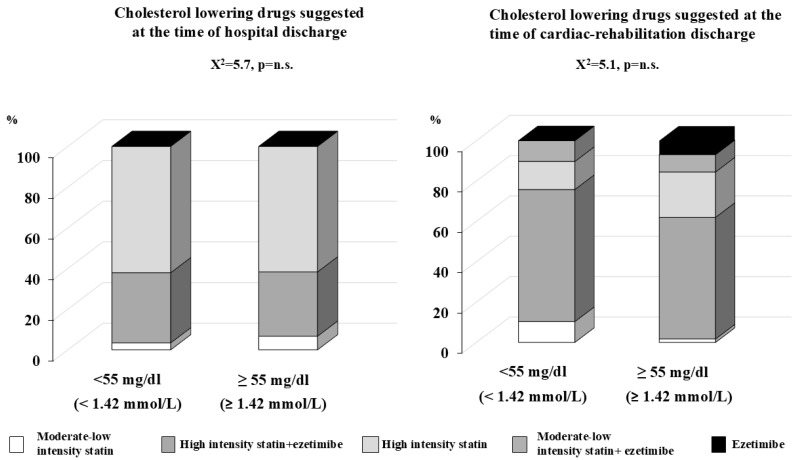
Lipid-lowering drugs suggested at the time of hospital discharge on the left panel and at the time of discharge form cardiac rehabilitation (right panel) among group 1 patients (n = 118); patients admitted to hospital for their first cardiovascular event, divided into two subgroups: those who reached and those who did not reach the LDL target < 55 mg/dL (1.42 mmol/L).

**Figure 3:**
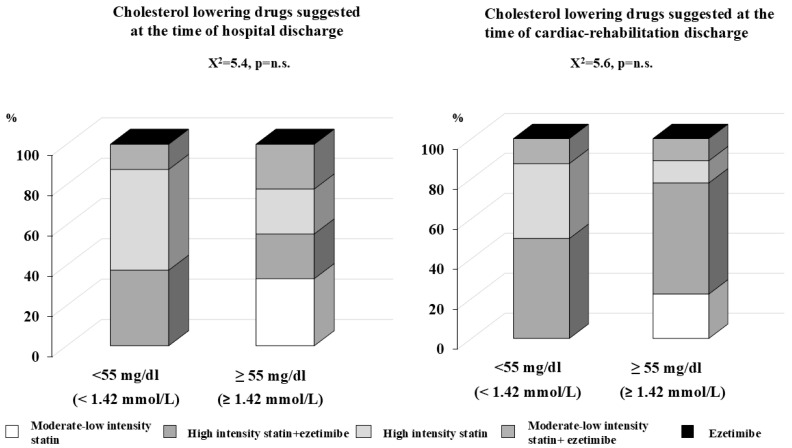
Lipid-lowering drugs suggested at the time of hospital discharge on the left panel and at the time of discharge form cardiac rehabilitation, right panel, among group 2A patients (n = 18); patients admitted to hospital for a second cardiovascular event 2 years after the previous one, divided into two subgroups: those who reached and those who did not reach the LDL target < 55 mg/dL (1.42 mmol/L).

**Figure 4:**
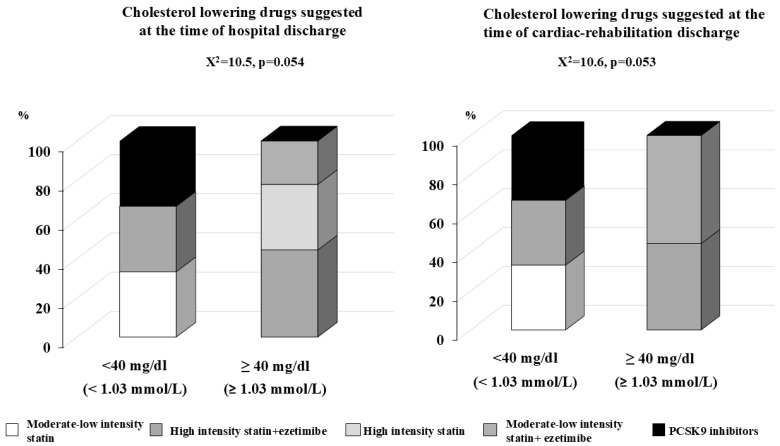
Lipid-lowering drugs suggested at the time of hospital discharge on the left panel and at the time of discharge form cardiac rehabilitation, right panel, among group 2B patients (n = 19); patients admitted to hospital for a second cardiovascular event within 2 years of the previous one, divided into two subgroups: those who reached and those who did not reach the LDL target < 40 mg/dL (1.03 mmol/L).

Group 1 presented a higher level of LDL cholesterol at admission to the hospital and presented a significant (*p* < 0.001) and progressive decrease in LDL levels during the observation period, while the changes in LDL cholesterol for groups 2A and 2B were less evident. The percentage of patients who reached the LDL target according to the guidelines was low in each of the three groups: 32.6%, 47.1%, and 25%, for groups 1, 2A, and 2B, respectively. Lipid-lowering drugs employed to reach the appropriate LDL goal according to the CV risk profile, as suggested by the guidelines, at the time of hospital discharge and at the time of discharge from cardiac rehabilitation are reported in Figure 2 for group 1, Figure 3 for group 2A, and Figure 4 for group 2B. Of note, among group 1 subjects, among those who reached and who did not the LDL goal, the vast majority was treated with high-intensity statin alone (62.1% and 61.7%, respectively), and only a small percentage was treated with high-intensity statin plus ezetimibe (34.5% and 31.6%, respectively). In the same group, after being discharged from cardiac rehabilitation, the percentage of those treated with high-intensity statin plus ezetimibe increased to 65.5% for those who reached the target and up to 60.3% for those who did not. Moreover, after discharge from cardiac rehabilitation, the percentage of those treated with moderate–low-intensity statin alone or plus ezetimibe also increased, and no one was prescribed PCSK9 inhibitors (Figure 2: *p* value for comparison is not statistically significant). Examining the results for group 2A (Figure 3), among those who reached the LDL level both after discharge from hospital or after cardiac rehabilitation, there was a higher percentage of patients treated with high-intensity statins alone or plus ezetimibe, even if the differences were not statistically significant; among those who did not reach their LDL goal, the were patients treated with moderate–low-intensity statin alone or plus ezetimibe. Again, none received PCSK 9 inhibitors. Among those who experienced a second CV event within 2 years of the previous one (group 2B), the most ambitious target of LDL < 40 mg/dL (<1.03 mmol/L) was reached both after hospital discharge and cardiac rehabilitation discharge, thanks to the prescription of PCK9 inhibitors (33.3% in both groups). The rest of the subjects were treated with high-intensity statin plus ezetimibe, and only one third with moderate–low-intensity statins. Among those who did not reach the target at the time of hospital discharge, less than half (44.4%) were treated with high-intensity statin plus ezetimibe, and at the time of discharge from cardiac rehabilitation, the majority (55.6%) was treated with moderate–low-intensity statin plus ezetimibe (*p* value for comparison among those who reached and who did not reach their LDL goals after hospital discharge (0.054), and a comparison of the subgroups after cardiac rehabilitation discharge (0.053)) (Figure 4).

### Clinical Audit

During the clinical audit, the reasons for the lack of achievement of the LDL goal were discussed with clinicians. According to the medical records available, the hypotheses presented were:

The general practitioner had changed the prescription suggested by the specialist at the time of discharge from hospital or the patient may not have shown to the general practitioner the letter of discharge, and for this reason, the appropriate prescription of the correct lipid-lowering drugs was not performed.

Muscle pain was another reason why statin intensity was reduced or the lipid-lowering prescription was not administered at the proper dose.

Drug adherence is another reason. It was observed that the patient did not take the proper dose of drugs or at the correct frequency, or they did not adhere to the hypo-lipidemic diet suggested during cardiac rehabilitation.

Moreover, clinical inertia of the specialist to prescribe the appropriate dose and type of drugs according to the LDL level of the patients at the time of admission to hospital or to cardiac rehabilitation was observed.

During the clinical audit, the instruments that could make it easier for the clinicians to identify the proper LDL target and the drugs useful to achieve it were also discussed (Appendix B Table A2, Table A3, Table A4 and Table A5). Table A2 presents a calculator that easily identifies the baseline LDL level of the patients, considering the LDL goal, and the percentage of LDL reduction required to obtain it. The second calculator, shown in Table A3, helped to identify the baseline LDL levels of the patients, considering the percentage of reduction required, and the LDL goal. Table A4 presents the calculator that provided information to the clinicians regarding the percentage of LDL reduction achieved by the patients, considering the baseline LDL level of the patients, and the level reached with treatment. And, finally, Table A5 sums up the percentage of LDL reduction that can be obtained according to different lipid-lowering drugs [5].

## 4. Discussion

This study explored the appropriateness of prescription of lipid-lowering drugs in a group of secondary prevention patients followed by cardiologists after an acute CV event. In contrast to Da Vinci’s [8] study, in our study, we did not include patients with other CV diseases, like cerebrovascular disease or peripheral artery disease, or patients in primary prevention care, as our patients enrolled in cardiac rehabilitation were all in secondary prevention care who experienced acute coronary heart disease (first episode or a recurrence within or after two years of the previous one). Similar to what was previously found in the literature [8,9], at the time of admission to the hospital, subjects both following their first CV event or those with a recurrence of the event, presented a high level of LDL cholesterol. This finding reflects the underestimation of the CV risk profile, similar to what was also observed in Santorini’s study [9], as, according to the risk score charts, in our study only 5.1% presented a low CV risk profile, while the rest were at a high or very high risk, and the vast majority (81.7%) was untreaded with lipid-lowering drugs, indicating a misperception of the risk of these patients.

According to the European data [8,9,16,17], also in our study, less than half reached the appropriate LDL goal; the percentage rose to 50% only among those who experienced a second CV event, but 2 years after the previous one (47%), probably due to the increased attention paid by the physicians in order to reduce the CV risk profile (87.5% were treated with high-intensity statin alone or in combination with ezetimibe both at the time of hospital discharge or cardiac rehabilitation discharge). Only one quarter of the subjects in group 2B achieved the more ambitious goal of <40 mg/dL (<1.03 mmol/L), and the achievement of the goal was due thanks to the adjunct of PCSK9 inhibitor. The relevance of LDL reduction is of crucial importance also for the recurrence of acute myocardial infarction, as documented by Ciliberti et al. [18]. The authors investigated a peculiar group of patients affected by acute myocardial infarction with non-obstructed coronary arteries and observed that, among those who needed to undergo a new invasive coronary angiography for the recurrence of an acute event, only 4.5% reached the recommended LDL target level. The most recent literature shows that the risk of a CV event is not only directly correlated to LDL level (as we know that a 1 mmol/L LDL reduction leads to a 21% CV risk decrease), but also to time of exposure to high LDL cholesterol levels [19,20]. So, the importance not only to achieve the appropriate goal but also to reach it as early as possible becomes clear. According to this evidence, the Italian Society of Interventional Cardiology (GISE) published a position paper [21] identifying which kinds of patients can benefit from an earlier (in-hospital or discharge) prescription of PCSK9 inhibitors. Similarly, Ray et al. [22] subsequently described a procedure for an earlier and more intensive prescription, according not only to the risk profile, but also to angiographic characteristics and individuals’ baseline LDL levels. The favorable effect of PCSK9 inhibitors is due not only to the mere reduction in LDL levels, but also to the reduction in the progression of atherosclerotic disease, with a stabilizing and reducing effect on atheromatous plaque, documented by an increase in the thickness of the fibrous cap and a reduction in its lipid arc [5,23]. Similar to previous findings [8,9], our data highlight the possibility to optimize lipid-lowering therapy prescriptions, implementing the use of combination therapy or the adjunct of PCSK9 inhibitors. The real-world observational study, HALES [24], clearly demonstrates the required primary and secondary prevention of the adjunct of PCSK9 inhibitors to easily reach the appropriate and necessary LDL target. During 12 months of follow up, the percentage of subjects that reached the goal of <1.8 mmol/L (<70 mg/dL) increased from 7.9% to 69.8%, and for the more ambitious target < 1.4 mmol/L (<55 mg/dL), it increased from 4.4% to 57.8% [24]. In our study, the percentage of combination therapy was below <50% both in patients that experienced their first CV event or their second CV event; of note, it increased up to ≥50% after discharge form cardiac rehabilitation. Moreover, PCSK9 inhibitors were underused and reserved only for patients that experienced a second CV event within 2 years of the previous one, probably due to the more ambitious goal required (<40 mg/dL (<1.03 mmol/L). Clinicians may have perceived this group of patients as at an increased risk and were more prone to prescribe high-intensity drugs, as well as patients being more adherent. The possible causes for this underprescription may be due to the local guideline recommendations and reimbursement constraints, similar to what was also reported by Asian colleagues in the HALES study [24]. In our country, the prescription of these drugs is restricted to patients that experience a CV event and, despite the combination of statin at the highest dosage tolerated plus ezetimibe or ezetimibe alone, if statin causes adverse effects, patients still have an LDL level ≥ 100 mg/dL (≥2.58 mmol/L). Only from 2022, the Agenzia Italiana del Farmaco reduced the cut off level for the reimbursement to ≥70 mg/dL (1.81 mmol/L) [25]. Of note, at the time of our study, both the GISE document [21] and the update of the Agenzia Italiana del Farmaco [25] were not yet available. Moreover, in our study, information about bempedoic acid was lacking. This drug [26,27] showed an additional reduction of 19.2 mg/dL (0.5 mmol/L) of LDL level in comparison to the placebo (*p* < 0.001), during 12 weeks of follow up, among patients who had an LDL level of 70 mg/dL (1.81 mmol/L) with or without lipid-lowering therapy [25]; and among patients with hypercholesterolemia and at high CV risk profile treated with stable lipid-lowering therapy, bempedoic acid led to a percentage change in LDL level of −23.0% after 12 weeks of treatment in comparison to the placebo (−1.5%, *p* < 0.001) [26]. Of note, at the time of our study, this drug was not yet available in Italy, as the Agenzia Italiana del Farmaco published the criteria for the reimbursement in 2023 [28]. According to the literature, all lipid-lowering drugs play an important role in reducing CV risk burden thanks to the reduction in LDL levels, but some of them reduce inflammation too. In an elegant review of a randomized controlled trial of lipid-lowering therapy, Iannuzzo et al. [29] highlighted the safety and efficacy of ezetimibe and of the newest drugs (bempedoic acid, alirocumab, and evolocumab) for preventing the development of major adverse CV events due to effectiveness of LDL reduction without an association to statins, and for two of them (bempedoic acid and ezetimibe), this was also due to a significant decrease in the high-sensitivity C-reactive protein, a well-known marker of the inflammation process.

In comparison to Da Vinci’s [8] and Santorini’s [9] studies, our study performed a critical revision of the process of prescription during clinical audits. During the clinical audit, we speculated about the main causes of the under prescription or improper prescription of lipid-lowering drugs, such as poor adherence, clinical inertia, muscle pains, and decisions of the general practitioner. These results emphasize some systematic problems in our healthcare system, such as a lack of awareness by clinicians and, in particular, by general practitioners; a lack of awareness of the updated guidelines; the high costs of the drugs, especially of PCSK9 inhibitors, which could be a deterrent for their prescription; and the reluctance of the patients to take high-intensity drugs due to the possible adverse effects, similar to what was found in previous studies [8,9,24]. Moreover, our clinical audit gave us the opportunity to develop some calculators that may help clinicians to identify the appropriate drugs and dosages to achieve the right LDL goal and percentage reduction according to individual risk profiles, starting from the individual baseline LDL level. The final phase of the clinical audit, which is still ongoing, will tell us if these calculators may be useful to ameliorate the appropriateness of lipid-lowering drug prescriptions.

### Limitations

Our study is a retrospective analysis, and we could not evaluate the adherence to medications and control of other risk factors that included routine care and patient choices or established cause–effect relationships. Moreover, as it was based on medical records, we did not have all the information regarding the reasons why general practitioners changed the prescribed drugs or the percentage of patients that experienced muscle pains.

The number of patients investigated was small, but we have to take into account that the years explored in this analysis were during the COVID-19 pandemic, with restrictions on the number of patients that had access to cardiac rehabilitation due to spatial restriction rules.

The small number of study participants did not provide us the opportunity to investigate the LDL goals achieved with each single drug and dosage. Moreover, because of the reduced number of female subjects, we cannot extend our results to the general population. The final limitation was the lack of information regarding subsequent adherence to diet and to the program of physical activity individually suggested during cardiac rehabilitation. The importance of the adherence to non-pharmacological treatment, especially to perform regular physical activity, is very important, however, usually the adherence to exercise is poor also according to the literature [30,31]. A possible solution for adhering to the suggestions may be the use of telemedicine and, for long-term outpatients, telemonitoring [32,33,34].

## 5. Conclusions

Our retrospective study highlighted the under prescription of lipid-lowering drugs both in terms of the types of drugs and dosage of each drug. According to our clinical audit, the lack of appropriateness was probably due to different causes, including poor patient adherence to drugs, diet and physical activity, clinical inertia, adverse effect of statins, like muscle pain, and possible modifications to the prescribed therapy administered by general practitioner. The last phase of the clinical audit will tell us if the calculators developed will help to ameliorate the appropriateness of the prescription of lipid-lowering drugs.

## Figures and Tables

**Table 1:** Moderate–low-intensity and high-intensity statin classifications.

Statin	Intensity
Atorvastatin 80 mg Atorvastatin 40 mg	High
Atorvastatin 20 mg Atorvastatin 10 mg	Moderate-low
Rosuvastatin 40 mg Rosuvastatin 20 mg	High
Rosuvastatin 10 mg Rosuvastatin 5 mg	Moderate-low
Simvastatin 40 mg Simvastatin 20 mg	Moderate-low

**Table 2:** Baseline characteristics of the study participants at the time of hospital entry.

Variable	Total n = 155	Group 1 n = 118	Group 2A n = 18	Group 2B n = 19	Χ^2^
Age, years	66.1 ± 9.4	65.4 ± 9.7	68.7 ± 8.9	68.0 ± 8.0	n.s. *
Sex, males (%)	137 (88.4)	102 (86.4)	18 (100)	17 (89.5)	n.s.
Overweight/obesity (%)	112 (72.4)	86 (73.8)	12 (70.6)	13 (68.4)	n.s.
IFG/DM (%)	39 (63.9)	68 (57.8)	13 (73.0)	16 (88.5)	n.s.
Smokers (%)	58 (72.5)	82 (69.2)	15 (83.4)	16 (88.5)	n.s.
Hypertension (%)	93 (93.0)	107 (91)	18 (100)	19 (100)	n.s.
Dyslipidaemias (%)	88 (91.7)	103 (87.5)	18 (100)	19 (100)	n.s.

Data are presented as average ± standard deviation for age; other variables are presented as numbers and (percentages). * *p* value expressed as ANCOVA analysis adjusted for sex. IFG, impaired fasting glucose; DM diabetes mellitus.

**Table 3:** Metabolic, biochemistry, and hemodynamic characteristics of the study participants at the time of hospital entry.

Variable	Total n = 155	Group 1 n = 118	Group 2A n = 18	Group 2B n = 19	*p* Value
BMI, kg/m^2^	28.9 ± 17.6	29.5 ± 20.1	27.9 ± 3.6	26.4 ± 2.9	n.s.
Hb g/dL	13.7 ± 1.6	13.8 ± 1.6	14.1 ± 1.2	12.7 ± 1.5	0.015
Serum glucose, mg/dL	110.5 ± 28.4	107.6 ± 25.2	110.5 ± 22.0	126.1 ± 43.8	n.s.
Serum creatinine, mg/dL	0.96 ± 2.3	0.96 ± 0.2	0.99 ± 0.2	0.95 ± 0.3	n.s.
TC, mg/dL (mmol/L)	181.6 ± 51.8 (4.69 ± 1.34)	191.1 ± 49.3 (4.94 ± 1.27)	162.9 ± 45.8 (4.21 ± 1.18)	137.9 ± 47.5 (3.56 ± 1.23)	<0.001
LDL-c, mg/dL (mmol/L)	115.8 ± 39.4 (2.99 ± 1.02)	122.8 ± 38.2 (3.17 ± 0.99)	101.7 ± 33.5 (2.63 ± 0.87)	81.4 ± 33.2 (2.1 ± 0.86)	<0.001
HDL-c, mg/dL (mmol/L)	43.8 ± 11.3 (1.13 ± 0.29)	45.2 ± 11.1 (1.17 ± 0.29)	40.5 ± 10.3 (1.05 ± 0.27)	38.8 ± 12.6 (1.0 ± 0.33)	n.s.
Tg, mg/dL	116.5 ± 54.6	112.7 ± 53.0	143.6 ± 60.1	109.9 ± 53.3	n.s. *
SBP, mmHg	142.0 ± 27.1	144.3 ± 28.8	143.3 ± 12.1	126.5 ± 15.6	n.s.
DBP, mmHg	82.2 ± 13.1	83.1 ± 13.1	85.0 ± 15.2	75.0 ± 10.8	n.s.

Data presented as average ± standard deviation. *p* value adjusted for age and sex. * *p* value for log-transformed data. BMI, body mass index; Hb, hemoglobin; TC, total cholesterol; LDL-c, low-density lipoprotein cholesterol; HDL-c, high-density lipoprotein cholesterol; Tg, trygliceride; SBP, systolic blood pressure; DBP diastolic blood pressure.

**Table 4:** LDL cholesterol level mg/dL (mmol/L) at the time of admission to hospital, at the start of cardiac rehabilitation, and at the first control visit within 1 year of follow up after rehabilitation.

Time of LDL Detection	Group 1	Group 2A	Group 2B	*p* Value for Group Comparison *
Admission to hospital	122.8 ± 38.2 (3.17 ± 0.99)	101.7 ± 33.5 (2.63 ± 0.87)	81.4 ± 33.2 (2.1 ± 0.86)	<0.001
Entry into rehabilitation	67.7 ± 26.9 (1.75 ± 0.7)	62.6 ± 17.4 (1.62 ± 0.45)	62.7 ± 22.0 (1.62 ± 0.57)	n.s.
Control visit	63.8 ± 20.8 (1.65 ± 0.54)	74.2 ± 26.4 (1.92 ± 0.68)	62.5 ± 19.3 (1.61 ± 0.5)	n.s.

* *p* value adjusted for age and sex.

## Data Availability

The original contributions presented in this study are included in the article. Further inquiries can be directed to the corresponding author.

## Appendix A

### Inclusion and Exclusion Criteria to Participate in Cardiac Rehabilitation

Inclusion criteria:

- Age > 18 years and ≤75 years.
- Discharge from cardiology unit after an acute atherosclerotic cardiovascular disease (see the complete list below).
- Ability to perform physical exercise.
- At least 20 days after discharge from hospital.

Exclusion criteria:

- Age > 75 years.
- Orthopedic disease that precludes performing physical activity.
- Denial of the patient to participate.
- Inability to attend the eight training sessions (i.e., family or working problems).

## Appendix B

### List of the Atherosclerotic Cardiovascular Diseases of the Patients That Participated in Cardiac Rehabilitation

Group 1 (n = 118 subjects): 75 patients had myocardial infarctions; 21 angina pectoris; 22 had an increase in cardiac serum markers during an acute event, such as heart failure, atrial fibrillation, acute infection, and valvular diseases; or patients who underwent coronary revascularization for the control of a known coronary artery disease.

Group 2 (2A n = 18, 2B n = 19 patients): 10 experienced a myocardial infarction; 13 had angina pectoris; and 14 presented and increase in cardiac serum markers during an acute event, such as heart failure, atrial fibrillation, acute infection, valvular diseases, and chronic coronary syndrome; or patients who underwent coronary revascularization to control a known coronary artery disease.

**Table A1:** Metabolic, biochemistry, and hemodynamic characteristics of the study participants at the time of hospital entry.

Variable	Total n = 155	Group 1 n = 118	Group 2A n = 18	Group 2B n = 19
BMI, kg/m^2^	28.9 (24.7, 29.5)	29.5 (24.8, 29.4)	27.9 (25.5, 29.7)	26.4 (24.0, 29.2)
Hb g/dL	13.7 (12.8, 14.8)	13.8 (12.8, 29.4)	14.1 (13.3, 14.9)	12.7 (11.7, 14.0)
Serum glucose, mg/dL	110.5 (90.7, 119.7)	107.6 (89.7, 117.2)	110.5 (97.0, 126.0)	126.1 (105.0, 124.0)
Serum creatinine, mg/dL	0.96 (0.80, 1.08)	0.96 (0.80, 1.05)	0.99 (0.83, 1.14)	0.95 (0.71, 1.13)
TC, mg/dL (mmol/L)	181.6 (144.0, 214.0) (4.69 (3.72, 5.53))	191.1 (156.5, 225.0) (4.94 (4.04, 5.81))	162.9 (131.0, 179.0) (4.21 (3.39, 4.63))	137.9 (109.2, 155.0) (3.56 (2.82, 4.01)
LDL-c, mg/dL (mmol/L)	115.8 (91.0, 144.0) (2.99 (2.35, 3.72))	122.8 (96.0, 152.2) (3.17 (2.48, 3.93))	101.7 (81.0, 114.0) (2.63 (2.09, 2.95))	81.4 (64.0, 96.0) (2.1 (1.65, 2.48))
HDL-c, mg/dL (mmol/L)	43.8 (36.0–50.7) 1.13 (0.93–1.31))	45.2 (38.0, 52.0) (1.17 (0.98, 1.34))	40.5 (34.0, 44.2) (1.05 (0.88, 1.14))	38.8 (33.0, 41.0) (1.0 (0.85, 1.06))
Tg, mg/dL	116.5 (78.2–144.2)	112.7 (73.5, 134.7)	143.6 (90.5, 177.2))	109.9 (81.0, 136.5)
SBP, mmHg	142.0 (128.0, 150.0)	144.3 (128.0, 153.0)	143.3 (130.0, 150.0)	126.5 (120.0, 140.0)
DBP, mmHg	82.2 (73.0, 90.0)	83.1 (80.0, 90.0)	85.0 (80.0, 100.0)	75.0 (70.0, 80.0)

Data presented as mean and (IQR). Abbreviations as listed in Table 1.

**Table A2:** Percentage of LDL reduction required to achieve the LDL goal < 55 mg/dL (1.42 mmol/L).

Percentage of LDL Reduction in Order to Obtain an LDL < 55 mg/dL		
Example of baseline LDL level mg/dL (mmol/L):	LDL goal mg/dL (fixed value):	Percentage of reduction required:
120 (3.1)	55 (1.42)	−54

**Table A3:** LDL value to achieve in order to obtain an LDL reduction ≥ 50%.

Value of LDL to Achieved to Obtain an LDL Reduction ≥ 50%		
Example of baseline LDL level mg/dL (mmol/L)	Target of LDL to achieve:	Percentage of LDL reduction (fixed value):
120 (3.1)	60 (1.55)	−50

**Table A4:** Effective percentage of LDL reduction obtained by the patient.

Percentage of LDL Reduction Obtained by the Patient		
Example of baseline LDL mg/dL (mmol/L):	Example of LDL level reached by the patient:	Percentage of LDL reduction obatined by the patient:
120 (3.1)	81 (2.09)	−33

**Table A5:** Percentage of LDL reduction according to different type of statins [5].

Type of Lipid-Lowering Drug	Percentage of LDL Reduction	Example of Drugs
High-intensity statins	about 50%	Atorvastatin 40–80 mg Rosuvastatin 20–40 mg
Moderate-intensity statins	30–50%	Atorvastatin 10–20 mg Rosuvastatin 5–10 mg Simvastatin 20–40 mg
Low-intensity statin	<30%	Simvastatin 10 mg
Ezetimibe as monotherapy	15–22%	
Ezetimibe plus any dose of statin	21–27% reduction above the level achieved by statin alone; 15% reduction when started as combination therapy in a naïve patient	
PCSK9 inhibitors	60% reduction (mean level according to different dosages)	
